# A New Treatment for Laryngeal Tuberculosis

**Published:** 1898-12

**Authors:** 


### A NEW TREATMENT FOR LARYNGEAL TUBERCULOSIS.

Leduc, of Nancy (*Munch. Med. Wochenschrift*), described before the recent Congress of the French Association for the Advancement of Science a method for the application of remedies to the larynx to be carried out by the patient himself. It consisted in the aspiration of certain medicaments in powdered form, and the author reported twenty-five cases in which recovery had been attained. The only instrument necessary is a glass tube with a caliber of six millimeters and a length of twenty to twenty-five centimeters. One centimeter distant from one end it is crooked at an angle of 100 degrees. The powder having been placed in the crooked end, the tube is introduced into the pharynx, and the patient then takes a short inspiration with the lips closed over the tube. For this method diiodoform alone, or where pain existed diiodoform mixed with cocaine or morphine, was employed. These aspirations were made from four to eight times a day. The author states that almost invariably patients who are quite aphonic, and who have the most pain on swallowing and the most serious dyspnea, show immediate improvement; the dyspnea subsides, the voice returns, and the patients can again take nourishment.

Leduc has used various powders, and has tried plain iodoform, which has usually so good an effect on tuberculous tissue, but with none has he had such excellent results as with diiodoform. His experience with this drug thus employed has led him to the conclusion that tuberculous laryngitis is one of the easiest of the symptoms of tuberculosis to alleviate.

